# Potential drug-drug interaction risks of selective serotonin reuptake inhibitors in outpatients: a retrospective analysis using two screening databases

**DOI:** 10.3389/fphar.2026.1965339

**Published:** 2026-10-21

**Authors:** Shanshan Xu, Zhihui Song, Yiman Li, Jie Bai, Dong Wang, Ente Wang, Jiawei Wang

**Affiliations:** Department of Pharmacy, Beijing Tongren Hospital, Capital Medical University, Beijing, China

**Keywords:** Lexi-Interact^®^, Micromedex, outpatients, potential drug-drug interactions, prevalence, selective serotonin reuptake inhibitors

## Abstract

**Introduction:**

Potential drug-drug interactions (pDDIs) involving selective serotonin reuptake inhibitors (SSRIs) pose significant risks in psychiatric care, where polypharmacy often exacerbates pharmacokinetic and pharmacodynamic effects through cytochrome P450 enzyme pathways. This retrospective study aimed to assess the prevalence, characteristics, and risk factors of pDDIs associated with SSRIs in outpatients.

**Methods:**

We conducted a retrospective study on outpatients admitted to Beijing Tongren Hospital from January 2023 to December 2024. The patients were ≧18 years old and treated with two or more drugs including at least one SSRI. Micromedex® and Lexi-Interact® were used to identify pDDIs and their severities, and logistic regression was employed to identify demographic predictors such as gender, age, and number of concomitant medications.

**Results:**

A total of 3476 prescriptions involving 1186 patients were included in the analysis. A prevalence of 19.68% was observed, with an average of 2.05 pDDIs per affected prescription. 98.83% of interactions detected by Micromedex® were classified as major, whereas among those detected by Lexi-Interact®, 68.76% were classified as moderate and 21.50% as minor. Agreement between the two databases on severity was poor (Cohen’s κ = 0.034) when the same drug pairs were rated by both. Common interacting drug classes included antipsychotics (292 instances), other antidepressants (207), blood glucose-lowering agents (207), antithrombotic agents (193), and calcium channel blockers (157). Predominant clinical consequences encompassed QT interval prolongation (569 cases), serotonin syndrome (390), bleeding (229), and hypoglycemia (207), aligning with serotonergic excess and platelet function theories. Female gender, middle age (40–64 years), and polypharmacy were significantly associated with higher pDDI risks.

**Conclusion:**

This retrospective study found pDDIs involving SSRIs relatively uncommon among general hospital outpatients, a broader, less-studied population than prior psychiatric or nursing home cohorts, yet still linked to severe outcomes, especially among female, middle-aged patients on polypharmacy. Discrepancies between interaction-severity databases underscored the need for multi-tool assessment. Future prospective, multi-centre studies are needed to inform safer outpatient psychiatric prescribing.

## 1 Introduction

Potential drug-drug interactions (pDDIs) are defined as the co-administration of two or more interacting medications with the potential to cause adverse clinical outcomes (Uijtendaal et al., 2014). These interactions may result in increased drug toxicity, diminished therapeutic efficacy, or unintended enhancement of a drug’s effects (Holm et al., 2014). Selective serotonin reuptake inhibitors (SSRIs) are a class of medications commonly prescribed for the treatment of depression, anxiety, and obsessive-compulsive disorders (Edinoff et al., 2021). They have been extensively characterized regarding CYP-P450 isoenzymes inhibition and pDDIs (Edinoff et al., 2021; Jin et al., 2016; Brown, 2008). The N-demethylation of fluoxetine is catalyzed by CYP2D6, 2C9, and 3A4 (von Moltke et al., 1997; Margolis et al., 2000), while the metabolism of citalopram involves CYP3A4, 2D6, and 2C19 (Sangkuhl et al., 2011). For paroxetine, CYP2D6 mediates the demethylation of its methylenedioxy group (Bloomer et al., 1992), and the N-demethylation of sertraline is mediated by CYP2B6, 2C9, 2C19, 2D6, and 3A4 (Obach et al., 2005). The metabolism pathways of fluvoxamine are oxidative demethylation and oxidative deamination by CYP-2D6 and CYP-1A2 (Spigset et al., 1997; Spina et al., 2003). Given the considerable differences in the ability of SSRIs to inhibit individual CYP enzymes, understanding these variations and relevant pDDIs can help guide the selection of an appropriate compound for the individual patient.

The increased prevalence of depression in both the young and the elderly populations has led to the addition of antidepressants to complex medication regimens. Great care must be exercised when selecting SSRIs in a multi-drug treatment regimen. Previous studies indicated that the pDDIs in patients with psychiatric disorders are common, with the prevalence varying from 28% to 88% (Lai et al., 2019; Yalçın et al., 2021; Aburamadan et al., 2021; Ranković et al., 2024; Bačar Bole et al., 2023; Borges et al., 2017). A cross-sectional retrospective study conducted on outpatients with depression found that the prevalence of pDDIs reached 31% (Chen and Ding, 2023). The outcomes of pDDIs in patients with SSRIs can lead to serious clinical adverse effects (AEs), including diminished therapeutic efficacy, increased toxicity of one or more of the administered compounds, QT-interval prolongation, and serotonin syndrome, which is rare but potentially life-threatening (Badar, 2024).

Although psychotropic drug-related pDDI risk has been extensively evaluated across antidepressants as a broad class, few studies have specifically quantified the prevalence, severity distribution, and demographic risk factors of pDDIs for SSRIs, the most widely prescribed antidepressant subclass, in outpatient populations using systematic, multi-database screening methodology, leaving this specific epidemiological gap unaddressed. The objective of this study was to evaluate the occurrence and characteristics of pDDIs between SSRIs and other drugs in prescriptions among outpatients. To identify pDDIs between SSRIs and other medications administered in the outpatient prescriptions more comprehensively, we utilized the Lexi-Interact® and Micromedex® online databases simultaneously, which have demonstrated the highest performance in detecting pDDIs (Kheshti et al., 2016; Clauson et al., 2007). Building on our group’s prior comparative evaluation of five distinct drug-drug interaction screening tools for SSRIs, which focused on tool-level accuracy and agreement (Xu et al., 2025), the present study instead applies two of the most widely used commercial databases to a larger, independent outpatient prescription cohort to characterize the real-world prevalence, severity, and demographic risk factors of SSRI-related pDDIs.

## 2 Methods

### 2.1 Study design

We conducted a retrospective cross-sectional study on outpatients at Beijing Tongren Hospital. The study included all outpatients prescribed two or more systemic medications (medications that are taken orally or by injection subcutaneously, inhalationally, intramuscularly, or intravenously), including at least one SSRI, from January 2023 to December 2024. By this definition, topical, ophthalmic, and other non-systemic-route formulations were excluded by design. We included five SSRIs marketed in the United States and also in our hospital’s drug formulary: fluoxetine (FXT), paroxetine (PAR), sertraline (SER), escitalopram (ESC), and citalopram (CIT). We excluded all outpatients whose prescriptions contained only Chinese traditional medicines and SSRIs, due to limited knowledge of pDDIs between these two medications. For prescriptions that combined Chinese traditional medicines with other systemic Western medications alongside an SSRI (rather than a Chinese traditional medicine plus SSRI only), no herb-drug interaction screening was attempted; only Western drug-SSRI pairs were screened for these retained prescriptions. The institutional ethics committee of Beijing Tongren Hospital approved the research. Due to the study’s retrospective and non-interventional nature, informed consent was not applicable.

### 2.2 Screening of prescriptions for potential drug-drug interactions (pDDIs)

In this study, a “prescription” refers to a distinct medication-combination instance per patient rather than each individual dispensing or visit event. If a patient was issued an identical combination of medications across multiple visits, this recurring prescription and its associated pDDI(s) were counted only once, in both the prevalence denominator (the total prescription count used for prevalence estimates, n = 3,476) and the corresponding pDDI tally, so as to avoid inflating either count through repeat dispensing of an unchanged regimen. The two online databases, including Lexi-Interact® and Micromedex®, were used to identify pDDIs between SSRIs and other systemic medications administered in the prescription. The two databases identified all the pDDIs and categorized the severity of pDDIs. Micromedex® and Lexi-Interact® each apply their own native severity rating systems; to allow direct comparison between the two databases, both were harmonized into five corresponding severity categories (severe, major, moderate, minor, and none), as fully defined in Supplementary Table S1, Supplemental Digital Content (Severe = Contraindicated/X; Major = Major/D; Moderate = Moderate/C; Minor = Minor/B; None = Unknown/No known interaction). All severity results reported in this manuscript use this harmonized five-category system. Cohen’s kappa (κ) was used to quantify the agreement between the two databases. The screening and severity classification of pDDIs were performed independently by two pharmacists. Their assessments were subsequently compared to ensure accuracy and consistency. In cases of discrepancy or ambiguity, a third pharmacist with a senior professional title was consulted to adjudicate and reach a final consensus classification. To avoid overcounting, each unique pDDI was recorded only once per patient, even if identified across multiple visits. The active ingredients within compound preparations were analyzed individually. It should be noted that this pDDI screening was performed retrospectively by applying both databases to historical prescription records, independent of any real-time clinical decision support alerts that may or may not have fired at the point of order entry; consequently, the prevalence estimates reported here reflect pDDIs detectable by these two databases rather than pDDIs actually flagged to, or acted upon by, the prescribing clinician, and no data on alert override rates or alert fatigue were available for this analysis. The overall medication categories were analyzed according to AHFS Drug Information® 2023.

### 2.3 Statistical analyses

Data analyses were conducted using IBM SPSS® statistical software version 29.0. Categorical variables were presented as percentages and continuous variables as mean ± standard deviation (SD). Chi-square (χ^2^) test as a univariable analysis and logistic regression as a multivariable analysis were performed by a stepwise selection approach to identify the risk factors associated with identified pDDIs. In the univariable model, the predictor variables included gender, age, and number of concomitant drugs. The odds ratio (OR) and 95% confidence interval (CI) were calculated for each variable. The predictors of pDDIs were detected using multiple regression analysis. To assess potential collinearity among the retained predictors, variance inflation factors (VIFs) were calculated for the final multivariable model. A gender × number-of-concomitant-drugs interaction term was additionally tested to evaluate whether the effect of polypharmacy on pDDI risk differed by sex. Overall model fit was assessed using the Hosmer-Lemeshow goodness-of-fit test. P values less than 0.05 were considered statistically significant.

## 3 Results

### 3.1 Demographic characteristics

During the study period, a total of 3476 prescriptions, including 1186 outpatients, were included in this analysis. The mean age of the patients was 62.25 ± 17.76 years, with 12.11% aged 18–39, 37.92% aged 40%–64% and 49.97% aged 65 or older. The female gender was predominant (59.23%) in all patients of all prescriptions. The number of concomitant drugs was 2–3 in 89.79% of prescriptions, 4–6 in 9.61%, and ≧7 in 0.60%, respectively (Table 1). The most commonly used SSRIs were ESC (73.62%, 2559/3476), followed by SER (14.15%, 492/3476), CIT (7.00%, 243/3476), PAR (4.29%, 149/3476), and FXT (0.95%, 33/3476).

**TABLE 1:** Characteristics of the study subjects.

Characteristics	Total prescriptions, n (% of overall cohort)	Prescriptions with ≥1 pDDI, n (% within subgroup)
Gender		
Male	1417 (40.77)	240 (16.94)
Female	2059 (59.23)	444 (21.56)
Age (years)		
18–39	421 (12.11)	85 (20.19)
40–64	1318 (37.92)	330 (25.04)
≧ 65	1737 (49.97)	269 (15.49)
Number of concomitant drugs		
2–3	3121 (89.79)	622 (19.93)
4–6	334 (9.61)	172 (51.50)
≧7	21 (0.60)	13 (61.90)

### 3.2 pDDIs between SSRIs and concomitant drugs

A total of 684 prescriptions (19.68%, 684/3476) were identified with 1404 pDDIs, with an average of 2.05 pDDIs per prescription. The prevalence of pDDIs was 16.94% in males and 21.56% in females. The number of concomitant drugs was measured at 2–3 (19.93%), 4–6 (51.50%), and seven or more (61.90%); 20.19%, 25.04% and 15.49% were in the groups of aged 18–39, 40–64 and 65 or older, respectively (Table 1).

Micromedex® detected 939 pDDIs in 94 drug combinations, of which 4 (0.43%) were classified as severe, 928 (98.83%) as major, and 7 (0.75%) as moderate (Figure 1); no pDDIs were classified as Minor or None. Lexi-Interact® detected 1335 pDDIs with 137 drug combinations, of which 7 (0.52%) were classified as severe, 123 (9.21%) as major, 918 (68.76%) as moderate, and 287 (21.50%) as minor (Figure 1). The two databases exhibited markedly different severity distributions: Micromedex® was skewed toward major, whereas Lexi-Interact® was skewed toward moderate/minor. There were 856 pDDIs simultaneously identified in two databases. Severity concordance was assessed for the subset of pharmacologic classes summarized in Supplementary Table S2, Supplemental Digital Content (n = 58 drug-pair entries, of which 32 were identified by both databases). Among these 32 dually-identified pairs, severity classifications were concordant for only 5 (15.6%; Cohen’s κ = 0.034), indicating essentially no agreement beyond chance. All 27 discordant pairs followed the same directional pattern, with Micromedex® rating the pair as major (Major tier) while Lexi-Interact® rated the same pair as C (Moderate tier) or B (Minor tier); no instance of the reverse pattern was observed. Concordance varied by clinical consequence type, ranging from 6.2% (1/16) for pairs associated with QT interval prolongation alone to 37.5% (3/8) for pairs associated with other consequences, while none of the four pairs associated with combined QT prolongation and serotonin syndrome were concordant. By concomitant medication class, antipsychotic agents contributed the largest number of dually-identified pairs (n = 5) and the highest class-level concordance (40.0%, 2/5), whereas the remaining classes each showed 0%–50% concordance among their typically 1–2 dually-identified pairs. When weighted by the number of underlying prescriptions affected rather than by unique drug pair, concordant classifications accounted for only 141 of the 856 overlapping pDDI events (16.5%), closely matching the pair-level concordance rate. There were four pDDIs identified as contraindicated by Micromedex®, and seven pDDIs as a risk rating of X by the Lexi-interact®, including ESC + domperidone, ESC + Rasagiline, SER + fluconazole, and PAR + potassium chloride (IV) (Table 2). For different SSRIs, the total number of pDDI events relative to total prescriptions containing that SSRI was highest for FXT (78.79%, 26 pDDI events among 33 FXT-containing prescriptions), followed by ESC (44.70%, 1144 events among 2,559 prescriptions), SER (35.98%, 177/492), PAR (30.87%, 46/149), and CIT (4.53%, 11/243). This metric reflects the average pDDI burden per prescription within each SSRI subgroup rather than the proportion of prescriptions with at least one pDDI. The most common concomitant medication class involved in pDDIs was antipsychotic agents (292), followed by antidepressants (207), blood glucose-lowering agents (207), antithrombotic agents (193) and calcium channel blocking agents (157) as shown in Table 3, which is organized by concomitant medication class to detail the specific interacting drug pairs and severity ratings associated with each of these top classes. For interacting pairs where two potential clinical consequences are listed (e.g., olanzapine + ESC/CIT), the consequences shown represent the union of both databases’ outputs and are not necessarily listed identically by each database; for example, Lexi-Interact® lists both serotonin syndrome and QT interval prolongation for several olanzapine–SSRI pairs, whereas Micromedex® lists QT interval prolongation only for the same pairs. The most common potential clinical consequences were QT interval prolongation (569), serotonin syndrome (390), bleeding (229) and hypoglycemia (207) as shown in Table 4, which instead is organized by potential clinical consequence to detail which medication classes contribute to each of these top consequence types. Database-specific consequence attributions for all pairs are provided in Supplementary Table S2, Supplemental Digital Content.

**FIGURE 1:**
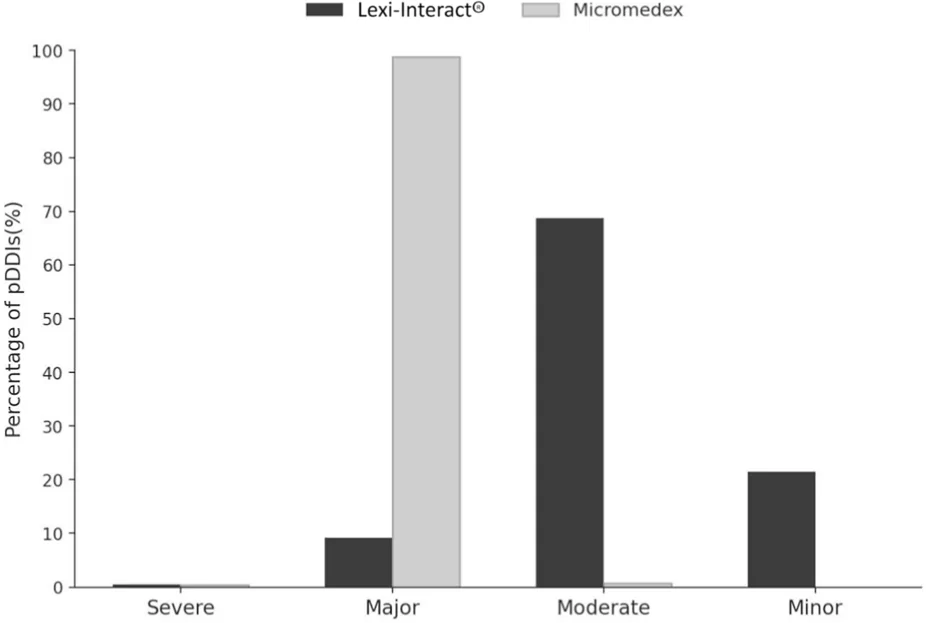
Severity of risk rating of potential drug-drug interactions by Micromedex® and Lexi-Interact®.

**TABLE 2:** Details of potential drug-drug interactions with risk rating severe by either database.

Interacting pairs	Number	Lexi-interact®	Micromedex®	Potential clinical consequence
ESC + domperidone	5	Severe	Major	QT interval prolongation
ESC + Rasagiline	2	Severe	Severe	serotonin syndrome
SER + fluconazole	1	Minor	Severe	QT interval prolongation
PAR + potassium chloride (IV)	1	Minor	Severe	Gastrointestinal irritation

**TABLE 3:** Description of the top five pharmacologic medications classes interacted with selective serotonin reuptake inhibitors (SSRIs), interactions pairs and severity in Micromedex^®^ and Lexi-Interact^®^, and the potential clinical consequence from interactions.

Concomitant medication classes	Interacting pairs	Number	Lexi-interact®	Micromedex®	Potential clinical consequence
Antipsychotic agents (292)	Olanzapine + ESC/FXT/CIT/SER	91	Moderate	Major	QT interval prolongation and serotonin syndrome
	Quetiapine + PAR/SER	17	Moderate	Major	QT interval prolongation
	Quetiapine + ESC	95	Major	Major	
	Flupentixol/Melitracen + ESC/CIT/SER	76	Moderate	Major	serotonin syndrome
	Flupentixol/melitracen + PAR/FXT	13	Major	Major	
Antidepressants (207)	Trazodone + ESC/FXT/CIT/PAR/SER	139	Moderate	Major	QT interval prolongation and serotonin syndrome
	Mirtazapine + ESC/SER	55	Moderate	Major	
	FXT + ESC	4	Moderate	Major	
	Duloxetine + ESC/FXT/PAR/SER	9	Moderate	Major	serotonin syndrome
Blood glucose- lowering agents (207)	All kinds of hypoglycemic agents + ESC/FXT/CIT/SER	207	Moderate	-	Hypoglycemia
Antithrombotic agents (193)	Rivaroxaban, edoxaban, dabigatran, heparin, clopidogrel, ticagrelor, aspirin + ESC/SER/CIT	192	Moderate	Major	Bleeding
	Cilostazol + ESC	1	-	Major	
Calcium channel blocking (CCB) agents (157)	Amlodipine, lacidipine, felodipine, nifedipine, nimodipine, diltiazem + ESC/FXT/CIT/PAR/SER	157	Minor	-	Fracture

**TABLE 4:** Description of the top five potential clinical consequence from interactions, medications classes interacted with selective serotonin reuptake inhibitors (SSRIs), interactions pairs and severity in Micromedex^®^ and Lexi-Interact^®^.

Potential clinical consequence	Number	Concomitant medication classes interacted with SSRIs	Number
QT interval prolongation	569	Antipsychotic agents	203
		Antidepressants	198
		Antibacterials	47
		Anti-dementia agents	38
		Antihistamines	28
		Adrenergics, inhalants	17
		Lipid modifying agents, plain	10
		Drugs for peptic ulcer and gastro-oesophageal reflux disease	9
		Urologicals	7
		Propulsives	7
		Cardiac preparations (others)	3
		Antifungals	2
serotonin syndrome	390	Antidepressants	298
		Antipsychotic agents	89
		Anti-Parkinson agents	2
		Antiepileptics	1
Bleeding	229	Antithrombotic agents	193
		Non-steroidal anti-inflammatory drugs	36
Hypoglycemia	207	Blood glucose-lowering agents	207

### 3.3 Risk factors associated with pDDIs

This study revealed significant differences between the prevalence of pDDIs within the group of gender (χ^2^ = 11.37, p < 0.001), age (χ^2^ = 180.28, p < 0.001), and the number of concomitant medications (χ^2^ = 200.79, p < 0.001). Univariable analysis showed that females (compared with males), patients aged 40–64, and those prescribed ≧7 concomitant drugs had the highest prevalence of pDDIs (Table 5).

**TABLE 5:** Association between patients’ characteristics and the risk of potential drug-drug interactions.

Variables	Chi-square test (univariable)		Multivariable logistic regression (adjusted)		
	Value	*p*-value	Adjusted OR (95% CI)	*p*-value	VIF
Gender	11.37	<0.001	​	​	​
Male	​	​	Reference	​	-
Female	​	​	1.48 (1.23–1.78)	<0.001	1.03
Age (years)	180.28	<0.001	​	​	​
18–39	​	​	Reference	​	-
40–64	​	​	1.33 (1.02–1.73)	0.036	1.65
≥65	​	​	0.73 (0.56–0.96)	0.023	1.78
Number of concomitant drugs	200.79	<0.001	​	​	​
2–3	​	​	Reference	​	-
4–6	​	​	4.31 (3.46–5.37)	<0.001	1.09
≧7	​	​	6.52 (2.73–15.58)	<0.001	2.12
Gender × concomitant-drug-count interaction (LR test)	-	-	χ^2^ = 6.91, df = 2	0.032	-
Female × 4–6 drugs (vs. Male × 2–3 drugs)	-	-	1.40 (1.24–1.95)	0.013	-
Female × ≧7 drugs (vs. Male × 2–3 drugs)	-	-	1.79 (1.21–2.74)	<0.001	-
Model fit: Hosmer-Lemeshow χ^2^ (df = 8)	-	-	8.40	0.396	-
Model fit: −2 log-likelihood/AIC/BIC	-	-	3112.34/3124.34/3161.27	-	-
Model fit: Nagelkerke R^2^/C-statistic (AUC)	-	-	0.146/0.707	-	-

The gender × drug-count interaction reported here was tested *post hoc* and was not part of the primary stepwise model; its clinical interpretation should be treated as exploratory and warrants confirmation in future studies.

Multivariable logistic regression indicated that female sex was associated with significantly increased odds of pDDIs (adjusted OR: 1.48, 95% CI: 1.23–1.78, *p* < 0.001). Patients aged 40–64 (adjusted OR: 1.33, 95% CI: 1.02–1.73, *p* = 0.036) had higher odds than patients aged 18–39, whereas patients aged ≥65 (adjusted OR: 0.73, 95% CI: 0.56–0.96, *p* = 0.023) had lower odds than patients aged 18–39. Patients prescribed 4–6 drugs (adjusted OR: 4.31, 95% CI: 3.46–5.37, ^p^ < 0.001) or ≥7 drugs (adjusted OR: 6.52, 95% CI: 2.73–15.58, *p* < 0.001) had higher odds of pDDIs than patients prescribed only 2–3 drugs. Variance inflation factors for all variables were below 3 (range: 1.03–2.12), indicating no problematic collinearity. The model showed acceptable discrimination (C-statistic = 0.707) and good calibration (Hosmer-Lemeshow χ^2^ = 8.40, df = 8, *p* = 0.396). A gender × concomitant-drug-count interaction term was additionally tested and reached statistical significance overall (likelihood-ratio test, χ^2^ = 6.91, df = 2, *p* = 0.032), indicating that the association between drug count and pDDI risk may differ somewhat by sex: relative to male patients prescribed 2–3 drugs, female patients prescribed 4–6 drugs had an additional OR of 1.40 (95% CI: 1.24–1.95, *p* = 0.013), and female patients prescribed ≥7 drugs had an additional OR of 1.79 (95% CI: 1.21–2.74, *p* < 0.001); given the exploratory nature of this interaction, it is reported in Table 5 but not further interpreted here.

## 4 Discussion

With the widespread global use of SSRIs, concerns about their safety have gradually emerged, especially the concern of drug interactions. This study reveals the prevalence of SSRI-related pDDIs in real-world outpatient prescriptions. The prevalence of pDDIs was 19.68% in our study. Rafi et al. concluded that 57.5% of the prescriptions had at least one pDDI associated with antidepressants in two major hospitals of Karachi, and the ratio of pDDI per prescription was 0.78 (Rafi et al., 2015). Liperoti et al. found the prevalence of potential antipsychotic drug interactions was 46.0% in impaired individuals aged 65 years or older residing in the participating nursing homes and being treated with antipsychotics (Liperoti et al., 2017). Zaabi et al. also concluded that the frequency of pDDIs among the psychiatric outpatients receiving antidepressant medications was 48.1% (Al Zaabi et al., 2021). The prevalence in the previous studies is generally higher than that in our study. We believe the narrower drug scope examined here is a principal driver of this difference, since our analysis was restricted to five SSRIs only, whereas the comparator studies cited above examined broader antidepressant classes (including serotonin-norepinephrine reuptake inhibitors, tricyclics, and/or monoamine oxidase inhibitors) that carry additional and often more extensive interaction potential. Differences in study population (hospitalized versus outpatient), drug interaction databases used, and criteria for defining pDDIs likely contributed further to this gap. Notably, this gap cannot be fully attributed to inpatient-versus-outpatient setting alone, since some of the higher-prevalence comparator studies cited above (Al Zaabi et al., 2021) were also conducted in outpatient populations. This distinction may be relevant here as well: unlike the cited comparator studies, which sampled residents of nursing homes (Liperoti et al., 2017) or patients attending psychiatric outpatient clinics (Al Zaabi et al., 2021), our cohort comprised all outpatients across a general hospital who were prescribed an SSRI alongside another systemic medication, without restriction to a psychiatric department or other specialized care setting. This unselected, hospital-wide sampling approach may better reflect SSRI use in a general outpatient population, whereas cohorts drawn from psychiatric clinics or long-term care facilities likely carry a higher burden of psychotropic polypharmacy and comorbidity, both established drivers of pDDI risk. To our knowledge, few prior studies of SSRI-related pDDIs have examined a similarly unselected, hospital-wide outpatient population, and we consider this an additional, underexplored contribution of the present study. Generally, it is widely established in the literature that antidepressant medications are frequently associated with clinically significant drug interactions.

Our study found that females, middle-aged individuals (40–64 years old), and those taking multiple concomitant medications were significant risk factors for pDDIs, which was aligned with previous studies (Rafi et al., 2015; Liperoti et al., 2017; Al Zaabi et al., 2021). However, an analysis of the FDA Adverse Event Reporting System to investigate the risks of serotonin syndrome among different SSRIs and assess the influence of drug-drug interactions with other medications also showed females and adults between 40 and 64 years old were those with the most reported cases (Novella et al., 2025). Women taking antidepressants are more likely to use them in combination with pain medications, gastrointestinal drugs, systemic hormones, and other agents, thereby increasing the risk of pDDIs (Mazza et al., 2024). Age-related trends revealed a peak in the 40–64 age group, declining in those ≥65 years, which may reflect cautious prescribing in the elderly due to heightened vulnerability to interactions involving CYP enzymes and comorbidities (Moura et al., 2009). Multivariate analyses in analogous studies have similarly identified polypharmacy as a primary risk factor, emphasizing the need for vigilant monitoring in multimorbid outpatients (Wang et al., 2022).

Discrepancies between Micromedex® and Lexi-Interact® were evident, with the former detecting 939 pDDIs (mostly major) and the latter 1335 (predominantly classified as moderate), yielding 856 overlapping interactions. Severity classifications of pDDIs in this investigation predominantly fell into major or moderate categories, with Micromedex® identifying 98.83% as major and Lexi-Interact® categorizing 68.76% as moderate. Such inconsistencies highlight the limitations of relying on a single database, as variations in severity classifications can influence clinical decision-making (Tragni et al., 2013). Beyond differences in severity rating, the two databases also diverged in their attribution of the specific clinical consequence for identical drug pairs (e.g., olanzapine-SSRI combinations, where Lexi-Interact® additionally flagged serotonin syndrome risk that Micromedex® did not report for the same pairs); clinicians relying on a single interaction-checking tool may therefore not only misjudge the severity of a given interaction, but may also fail to anticipate the specific adverse mechanism most relevant for monitoring.

In the presented study, the most common concomitant medication class involved in pDDIs with SSRIs was antipsychotic agents, which represented approximately 21% of total pDDIs, followed by antidepressants and blood glucose-lowering agents. This result is consistent with epidemiological patterns in psychiatric outpatients, where antipsychotics and other antidepressants frequently co-occur with SSRIs, contributing to 40%–50% of pDDIs due to shared serotonergic pathways (Chen and Ding, 2023; Holm et al., 2019). The most common medications that interacted with SSRIs were trazodone, quetiapine, olanzapine and flupentixol/melitracen. Previous studies have reported common interacting pairs associated with SSRIs. A study on predictors of interactions in psychiatry outpatients identified antipsychotics like quetiapine and olanzapine as common interactants, mirroring the QT prolongation risks observed here with escitalopram-quetiapine pairs (Novella et al., 2025). In contrast to pediatric cohorts, where antiepileptics and antihistamines predominate (Lai et al., 2019; Chen and Ding, 2023), the adult outpatient focus in this analysis emphasized cardiovascular agents, such as antithrombotics and calcium channel blockers, which align with multimorbidity in older age groups (49.97% aged ≥65) (Liu et al., 2023). Such categorizations support polypharmacy management strategies, advocating for deprescribing in high-risk classes to mitigate cumulative interaction burdens (Moura et al., 2009; Castilho et al., 2018). Moreover, prescriptions involving citalopram contributed the lowest pDDI rate. Citalopram is a weak *in vitro* inhibitor of CYP 2D6, and it has weak or no effects on CYP 1A2, CYP 2C19, and CYP 3A4 (Kobayashi et al., 1997). With respect to pharmacokinetic drug interactions, citalopram was not identified as the cause of clinically significant drug interactions in this cohort. This descriptive observation, however, was not derived from a formal statistical comparison of pDDI rates across SSRIs adjusted for confounders such as age and number of concomitant medications; citalopram’s comparatively low pDDI rate here is therefore consistent with, but does not by itself establish, a more favourable interaction profile relative to other SSRIs.

The most common potential clinical consequences identified were QT interval prolongation (569 instances), serotonin syndrome (390 instances), bleeding (229 instances), and hypoglycemia (207 instances), reflecting the pharmacodynamic and pharmacokinetic profiles of SSRIs. These outcomes corroborate literature on SSRI-related adverse events, where QT prolongation emerges as a leading concern in 30%–40% of pDDIs, particularly with concomitant antipsychotics or antifungals (Chen and Ding, 2023; Al Zaabi et al., 2021; Das et al., 2021). For instance, escitalopram-quetiapine interactions, rated D/major, were linked to QT risks, consistent with propensity score-matched studies associating SSRIs with cardiovascular events (Das et al., 2021). Serotonin syndrome, often precipitated by combinations like trazodone-escitalopram, aligns with reports of serotonergic excess in 20%–30% of psychiatric pDDIs (Chen and Ding, 2023; Desai et al., 2024). Bleeding risks, associated with antithrombotics like rivaroxaban-escitalopram, echo findings from elderly cohorts where SSRI-NSAID co-administration elevates haemorrhage odds by 1.2–12.4 (Al Zaabi et al., 2021). Hypoglycemia, tied to blood glucose-lowering agents, supports pharmacokinetic theories of SSRI-induced insulin sensitivity alterations (Lai et al., 2019; Holm et al., 2019). Although the interaction rating between calcium channel blocking (CCB) agents and SSRIs was only classified as B, a retrospective cohort study found that compared to CCB-only users, CCB-SSRIs users had a higher risk of fractures (hazard ratio: 1.43) (Desai et al., 2024). These consequences invoke the serotonin hypothesis and arrhythmogenic models, emphasizing clinical vigilance through electrocardiographic monitoring and dose adjustments (Das et al., 2021; Ismail et al., 2020). In the current analysis, contraindicated interactions, such as ESC with rasagiline (serotonin syndrome) or SER with fluconazole (QT prolongation), were rare but clinically critical, aligning with package inserts on avoiding such pairs to prevent life-threatening events.

Despite these insights, several limitations warrant consideration. First, the retrospective design, reliant on prescription data from a single institution, may underestimate actual interactions due to unrecorded over-the-counter medications or non-adherence, as noted in similar cross-sectional studies (Liu et al., 2023; Ren et al., 2020). Second, the exclusion of traditional Chinese medicines limits generalizability to diverse cultural contexts, potentially overlooking herb-drug interactions (Ren et al., 2020). Third, the severity assessments were confined to two databases, introducing classification biases. Fourth, and importantly, our pDDI estimates are based solely on database-defined interaction potential rather than confirmed clinical outcomes; we did not have access to patient-reported adverse reactions, chart-confirmed adverse events, or outcome-validation tools such as the Drug Interaction Probability Scale (Moura et al., 2009; van Leeuwen et al., 2013), and the true clinical impact of the pDDIs identified here therefore remains unquantified. Fifth, because pDDIs were identified retrospectively by applying Micromedex® and Lexi-Interact® to historical prescriptions rather than through prospective monitoring of clinical decision support alerts, we were unable to assess how many of these interactions were actually flagged to prescribers in real time, how alerts were acted upon, or the extent to which alert fatigue may have influenced prescribing behaviour; this remains an important open question for translating pDDI prevalence into actionable clinical impact. Sixth, the sample demographics, with a mean age of 62.25 years and female predominance (59.23%), may not fully represent younger or male-dominant populations (Lai et al., 2019; Holm et al., 2019). Last, since fluvoxamine was not in the drug list of our hospital, we have omitted any pDDIs associated with it. Future research should incorporate prospective designs with multi-centre data, integrating electronic health records for real-time interaction detection, alert-log analysis, and assessing actual adverse events (Wang et al., 2022; Ren et al., 2020). Additionally, because the pDDIs identified here reflect phenotypic drug-pair interactions as classified by commercial databases rather than genotype-informed risk, incorporating SSRI-specific pharmacogenomic factors, such as the CYP2C19 and CYP2D6 polymorphisms discussed in the Introduction, could help refine individual-level risk predictions beyond what drug-pair screening alone can offer (Al Zaabi et al., 2021; Liu et al., 2023). Benchmarking our findings against very large national-scale pharmacoepidemiologic datasets, such as a recent Iranian national cross-sectional study of over 2.3 million outpatient prescriptions (Aarabi et al., 2026), may also help contextualize our prevalence estimates against differently-scaled real-world cohorts. Similarly, a recent nationwide analysis of nearly nine million antidepressant prescriptions in Iran, which characterized prescribing trends by age, sex, and antidepressant class (Karami et al., 2025), and a study characterizing potentially inappropriate medication use by Beers criteria in older outpatients (Gholamnezhad et al., 2024), offer complementary large-scale evidence on outpatient prescribing safety that could inform future comparative analyses of our age- and sex-related findings. These directions hold promise for enhancing medication safety in outpatient psychiatry.

## 5 Conclusion

This retrospective study identified a 19.68% prevalence of pDDIs involving SSRIs in outpatient settings, with escitalopram and fluoxetine exhibiting the highest rates. Unlike prior studies focused on psychiatric outpatient clinics or nursing home residents, this cohort was drawn from all outpatients across a general hospital, offering a less-studied perspective on SSRI-related pDDI risk in a broader, unselected patient population. Key risk factors were female gender, middle age (40–64 years), and polypharmacy (≥4 drugs). The most frequent interacting drugs were antipsychotics, antidepressants, and antidiabetic agents, increasing risks of QT prolongation, serotonin syndrome, and bleeding. Discrepancies between Micromedex® and Lexi-Interact® databases underscored the value of multi-tool assessment for accurate severity classification. Future prospective, multi-centre studies incorporating pharmacogenomics and actual adverse event tracking are needed to enhance risk mitigation and inform clinical guidelines for outpatient psychiatry.

## Data Availability

The original contributions presented in the study are included in the article/Supplementary Material, further inquiries can be directed to the corresponding author.
