# Supplementary material for: Potential drug-drug interaction risks of selective serotonin reuptake inhibitors in outpatients: a retrospective analysis using two screening databases
Source: Front Pharmacol. 2026 Oct 21;17:1965339. doi: 10.3389/fphar.2026.1965339 (PMC13731344; doi:10.3389/fphar.2026.1965339)
Supplement: Supplementary file 1 [file Table1.docx]

**Supplemental Digital Content**

**Table S1.** Reclassification of potential drug-drug interactions severity in Micromedex^®^ and Lexi-Interact^®^

| **Reclassification of DDI severity category** | **Micromedex^®^** | **Lexi-interact^®^** |
| --- | --- | --- |
| Severe | Contraindicated (the drugs are contraindicated for concurrent use) | X. Avoid combination |
| Major | Major (interaction might be life-threatening and/or require medical intervention) | D. Consider therapy modification |
| Moderate | Moderate (interaction might result in exacerbation of the patient’s condition and/or requires an alternative therapy) | C. Monitor therapy |
| Minor | Minor (interaction might cause mild adverse effects. No clinical intervention is usually required; monitoring may be reasonable. Significant clinical consequences are very unlikely) | B. No action needed |
| Unknown | Unknown (no known drug interactions) | No known interaction |

**Table S2.** Description of the pharmacologic medications/classes interacted with SSRIs, interactions severity in Micromedex^®^ and Lexi-Interact^®^, and the potential clinical consequence from interactions

| **Concomitant medication classes** | **Interacting pairs** | **Number** | **Lexi-interact^®^** | **Micromedex^®^** | **Potential clinical consequence** | **Severity concordance***** |
| --- | --- | --- | --- | --- | --- | --- |
| Antipsychotic agents | Olanzapine+ESC/FXT/CIT/SER | 91 | Moderate | Major | QT interval prolongation and serotonin syndrome | Discordant |
|  | Quetiapine+PAR/SER | 17 | Moderate | Major | QT interval prolongation | Discordant |
|  | Quetiapine+ESC | 95 | Major | Major | QT interval prolongation | Concordant |
|  | Flupentixol/Melitracen+ESC/CIT/SER | 76 | Moderate | Major | serotonin syndrome | Discordant |
|  | Flupentixol/melitracen+PAR/FXT | 13 | Major | Major | serotonin syndrome | Concordant |
| Antidepressants | Trazodone+ESC/FXT/CIT/PAR/SER | 139 | Moderate | Major | QT interval prolongation and serotonin syndrome | Discordant |
|  | Mirtazapine+ESC/SER | 55 | Moderate | Major | QT interval prolongation and serotonin syndrome | Discordant |
|  | FXT+ESC | 4 | Moderate | Major | QT interval prolongation and serotonin syndrome | Discordant |
|  | Duloxetine+ESC/FXT/PAR/SER | 9 | Moderate | Major | serotonin syndrome | Discordant |
| Antiepileptics | Carbamazepine+ESC | 1 | Moderate | Major | serotonin syndrome | Discordant |
|  | Lamotrigine+ESC | 4 | - | Major | myoclonus | N/A (single database) |
|  | Lamotrigine+SER | 2 | - | Moderate | increased risk of lamotrigine toxicity (fatigue, sedation, confusion, decreased cognition) | N/A (single database) |
|  | Levetiracetam+ESC/FXT | 6 | Minor | - | psychomotor impairment | N/A (single database) |
| Lipid modifying agents, plain | Probucol+ESC | 9 | Moderate | Major | QT interval prolongation | Discordant |
|  | Probucol+SER | 1 | Minor | Major | QT interval prolongation | Discordant |
|  | Pravastatin+PAR | 3 | Moderate | - | hyperglycemia | N/A (single database) |
| Antibacterials | Levofloxacin, Moxifloxacin+ESC | 27 | Moderate | Major | QT interval prolongation | Discordant |
|  | Azithromycin+ESC | 7 | Moderate | Major | QT interval prolongation | Discordant |
|  | Azithromycin+PAR/SER | 4 | Minor | Major | QT interval prolongation | Discordant |
|  | Clarithromycin+PAR | 2 | Moderate | Major | QT interval prolongation | Discordant |
|  | Clarithromycin+SER | 1 | Minor | Major | QT interval prolongation | Discordant |
|  | Metronidazole+ESC | 5 | Minor | Major | QT interval prolongation | Discordant |
|  | Metronidazole+CIT | 1 | Moderate | Major | QT interval prolongation | Discordant |
| Anti-dementia agents | Donepezil+ESC/FXT | 29 | Minor | Major | QT interval prolongation | Discordant |
|  | Donepezil+SER | 9 | - | Major | QT interval prolongation | N/A (single database) |
| Antifungals | Fluconazole+ESC | 1 | Moderate | Major | QT interval prolongation | Discordant |
| Antihistamines | Loratadine, Desloratadine+ESC/CIT | 22 | Minor | - | QT interval prolongation | N/A (single database) |
|  | Ebastine+ESC/FXT/PAR | 6 | - | Major | QT interval prolongation | N/A (single database) |
| Adrenergics, inhalants | Budesonide/Fomoterol+ESC | 14 | Minor | Major | QT interval prolongation | Discordant |
|  | Budesonide/Fomoterol+PAR | 1 | - | Major | QT interval prolongation | N/A (single database) |
|  | Salmeterol/fluticasone+ESC | 2 | Minor | - | QT interval prolongation | N/A (single database) |
| Propulsives | Domperidone+SER | 2 | Minor | Major | QT interval prolongation | Discordant |
| Drugs for peptic ulcer  and gastro-oesophageal reflux disease | Famotidine+ESC/SER | 9 | - | Major | QT interval prolongation | N/A (single database) |
|  | CYP2C19 inhibitors (omeprazole, esomeprazole)+ESC | 13 | Moderate | Major | increased serum concentrations of SSRIs | Discordant |
| Cardiac preparations (others) | Ivabradine+ESC | 3 | - | Major | QT interval prolongation | N/A (single database) |
| Urologicals | Tolterodine+ESC | 3 | - | Major | QT interval prolongation | N/A (single database) |
|  | Solifenacin+ESC | 2 | Minor | Major | QT interval prolongation | Discordant |
|  | Solifenacin+SER | 2 | - | Major | QT interval prolongation | N/A (single database) |
| Blood glucose lowering agents | All kinds of hypoglycemic agents+ESC/FXT/CIT/SER | 207 | Moderate | - | hypoglycemia | N/A (single database) |
| Calcium channel blocking agents | amlodipine, lacidipine, felodipine, nifedipine, nimodipine, diltiazem+ESC/FXT/CIT/PAR/SER | 157 | Minor | - | fracture | N/A (single database) |
| Antithrombotic agents | rivaroxaban, edoxaban, dabigatran， heparin, clopidogrel, ticagrelor, aspirin+ESC/SER/CIT | 192 | Moderate | Major | bleeding | Discordant |
|  | Cilostazol+ESC | 1 | - | Major | bleeding | N/A (single database) |
| Non-Steroidal Antiinflammatory Agents | Nonselective (ibuprofen,nabumetone,diclofenac)+ ESC/PAR/SER | 28 | Major | Major | bleeding | Concordant |
|  | COX-2 Selective (celecoxib, etoricoxib)+ESC/SER | 8 | Moderate | Major | bleeding | Discordant |
| Peripheral vasodilators (CYP 2D6 substrates) | Nicergoline+ESC | 15 | - | Major | increased serum concentrations of substrates | N/A (single database) |
|  | Nicergoline+PAR | 1 | Moderate | - | increased serum concentrations of substrates | N/A (single database) |
| β-blocking agents (CYP 2D6 substrates) | Metoprolol+SER | 4 | Minor | Major | increased serum concentrations of substrates | Discordant |
|  | Metoprolol+PAR | 3 | Moderate | Moderate | increased serum concentrations of substrates | Concordant |
| α-adrenoreceptor antagonists (CYP 2D6 substrates) | Tamsulosin+ESC/SER | 14 | - | Major | increased serum concentrations of substrates | N/A (single database) |
|  | Tamsulosin+FXT | 2 | Moderate | Moderate | increased serum concentrations of substrates | Concordant |
|  | Tamsulosin+PAR | 1 | Moderate | Major | increased serum concentrations of substrates | Discordant |
| Potassium | Potassium chloride (IV)+ESC | 9 | Minor | - | hyponatremia | N/A (single database) |
| Thiazide-like diuretics | Hydrochlorothiazide+ESC, PAR | 17 | Moderate | - | hyponatremia | N/A (single database) |
|  | Indapamide+ESC | 2 | Moderate | - | hyponatremia | N/A (single database) |
| Thyroid agents | Levothyroxine+ESC/SER | 19 | Moderate | - | decrease therapeutic effects | N/A (single database) |
| Antivertigo agents | Flunarizine+ESC/FXT/PAR/SER | 17 | Minor | - | psychomotor impairment | N/A (single database) |
| Analgesics | Pregabalin+ESC/PAR | 3 | Minor | - | psychomotor impairment | N/A (single database) |
| Muscle relaxants | Baclofen+ESC/FXT | 5 | Minor | - | psychomotor impairment | N/A (single database) |

**Severity concordance was assessed for the subset of drug pairs identified by both databases (n = 32/58 pairs listed), using the reclassified Micromedex®–Lexi-Interact® severity correspondence defined in Table S1 (Severe = Contraindicated/X; Major = Major/D; Moderate = Moderate/C; Minor = Minor/B). Pairs identified by only one database are labelled “N/A (single database)” and excluded from the concordance calculation.*
